# Mapping climate suitability index for rainfed cultivation of medicinal plants by developing an AI-based probabilistic framework

**DOI:** 10.1038/s41598-024-71208-6

**Published:** 2024-09-02

**Authors:** Sina Sadeghfam, Mohammad Sina Rahmani, Marjan Moazamnia, Mohammad Reza Morshedloo

**Affiliations:** 1 Department of Civil Engineering, Faculty of Engineering, University of Maragheh, P.O. Box 55136-553, Maragheh, East Azerbaijan Iran; 2 Department of Civil Engineering, Faculty of Engineering, University of Bonab, Bonab, East Azerbaijan Iran; 3 Department of Horticultural Science, Faculty of Agriculture, University of Maragheh, Maragheh, East Azerbaijan P.O. Box 55136-553, Iran

**Keywords:** Agro-climate, Frequency analysis, Lake Urmia, Particle swarm optimization, Rainfed agriculture, Agroecology, Hydrology

## Abstract

The Climate Suitability Index (CSI) can increase agricultural efficiency by identifying the high-potential areas for cultivation from the climate perspective. The present study develops a probabilistic framework to calculate CSI for rainfed cultivation of 12 medicinal plants from the climate perspective of precipitation and temperature. Unlike the ongoing frameworks based on expert judgments, this formulation decreases the inherent subjectivity by using two components: frequency analysis and Particle Swarm Optimization (PSO). In the first component, the precipitation and temperature layers were prepared by calculating the occurrence probability for each plant, and the obtained probabilities were spatially interpolated using geographical information system processes. In the second component, PSO quantifies CSI by classifying a study area into clusters using an unsupervised clustering technique. The formulation was implemented in the Lake Urmia basin, which was distressed by unsustainable water resources management. By identifying clusters with higher CSI values for each plant, the results provide deeper insights to optimize cultivation patterns in the basin. These insights can help managers and farmers increase yields, reduce costs, and improve profitability.

## Introduction

Extending agricultural species with medicinal and commercial values can provide new opportunities for local communities to increase efficiency in agriculture^1^. The limitation in water and soil resources, pressure on natural ecosystems, climate change, drought, land use change, and increasing cultivation on slopes highlight the necessity for paying attention to sustainable agriculture^2^. Various strategies and approaches, including low-input, ecological, and conservation agriculture, have been developed to achieve sustainable agricultural systems^3^. Low-input agriculture aims to identify areas suitable for low-water-demand medicinal plants, replacing high-water-consumption crops.

Identifying areas with appropriate climate requirements and ecological environments is beneficial for cultivating high-quality medicinal plants and increasing the efficiency of agriculture. Soil, precipitation, and temperature are variables that affect the growth and quality of medicinal plants. While all these variables are important, soil characteristics change insignificantly over time, making external climate factors like precipitation and temperature more influential on the long-term growth dynamics of medicinal plants^4^. Different studies were undertaken to investigate the Climate Suitability Index (CSI), which reflects the the adaptivity degree of the climatic conditions in a study area for a specific type of crop (for example, see^5^). The literature review emphasizes indices known as CSI, which play a crucial role in shaping biodiversity. These indices have various applications in various fields, such as agriculture. However, the results are on a large scale and provide a general overview of agriculture without considering specific types of cultivation.

The common components for calculating cultivation potential are the incorporated data layers and overlaying techniques, summarized in Table 1 for a set of previous studies together with the plant name and study area. The table shows that different data layers are incorporated, which can be categorized into climatic variables (e.g., precipitation, temperature, and humidity), soil quality (e.g., pH, EC), and basin physiography (e.g., slope and altitude). The table also compared the studies from the point of view of overlaying techniques. The critical issue in this part is the relative importance of the incorporated data layers, referred to as the weights. The Analytical Hierarchical Process (AHP) is the most popular overlaying technique, which calculates the weights based on expert judgment using a pairwise comparison matrix. Other incorporated techniques are also relying on expert judgment. Notably, subjectivity can lead to inconsistencies in the outcomes, making the reproducibility of results difficult. Including subjective elements in modeling can also make it challenging to interpret the results, and it may not be transparent due to the influence of personal biases.

**Table 1:** List of previous studies from different point of views.

Row	Data layers	Overlaying techniques	Plant	Study area	References
1	Average/Min/Max annual temperature, Altitude, Soil covariates	Boolean and fuzzy logic	Rapeseed	Turkey	^6^
2	Precipitation, temperature, slope, altitude, soil texture, slope direction	AHP	Astragalus hypsogeton bunge	Iran	^6^
3	Land surface temperature, bareness of land, slope, surface elevation, humidity and rainfall	Equal weight overlap analysis	Jatropha curcas	Pakistan	^7^
4	Temperature, precipitation, humidity, slope, altitude, evaporation, slope direction, pH	AHP	Saffron	Iran	^8^
5	Soil parameters including, CaCO_3_, EC, PH, Slope, Texture	AHP	Rainfed wheat	Turkey	^9^
6	Average temperature in the flowering period, average temperature in the winter season, average annual relative humidity, average annual rainfall, average daily sunny time, altitude, slope, land use capability	AHP	Citrus	Turkey	^5^
7	Annual average temperature and precipitation, slope, slope direction, altitude, land use, soil texture	Weighted linear combination	Rainfed Chickpea and Irrigated Chickpea	Iran	^10^
8	Average isothermal temperature, seasonal temperature, annual rainfall and seasonal rainfall, evaporation	Maximum entropy model (Maxent)	Amorpha canescens, Leucaena collinsi, Cynodon dactylon, Atriplex canescens (Pursh)	Mexico	^11^
9	Temperature, precipitation	Maxent	Ephedra sinica	China	^12^
10	19 climatic and edaphic factors	Maxent	Cuphea aequipetala Cav	Mexico	^13^
11	Four bioactive compounds (madecassoside, asiaticoside, madecassic acid, and asiatic acid)	–	Centella asiatica	India	^14^
12	Temperature and precipitation	Entropy Catastrophe Scheme	Wheat	Iran	^15^

 Some overlaying techniques, such as the maximum entropy model, decrease the inherent subjectivity by modeling the geographic distribution of species with presence records and determining the distribution through the maximum entropy probability^11^. Also, the ensemble ecological niche model predicts the ecological position of a species by determining the most vital environmental elements^14^ and the weighted linear combination, which assigns a weight to each layer based on its importance^5^.

Unsupervised learning algorithms for cluster analysis employ unstructured data that are grouped based on similarities and patterns. Examples of these algorithms are the genetic algorithm^16^, density-based spatial clustering of applications with noise, and Particle Swarm Optimization (PSO)^17^. PSO was incorporated in previous studies as an optimizer model, e.g., mapping and protecting agricultural land under spatial constraints^18^ and determining soil nutrient management areas^19^. This study used PSO to combine precipitation and temperature layers to decrease the inherent subjectivity. PSO offers several advantages over other clustering techniques, including the ability to efficiently handle high-dimensional data, simplicity of implementation, and quick convergence to a solution^20^. Additionally, PSO is less likely to be trapped in local optima compared to other optimization techniques^21^. Notably, the study does not aim to evaluate the clustering or optimization techniques; further techniques are available in the literature^22–24^.

In addition to the economic benefits, the rainfed cultivation of medicinal plants can take a significant step toward restoring the distressed Lake Urmia. This lake, located in the northwest of Iran, experienced a state of distress regarding water resource unsustainability^25,26^, and it lost 96% of its surface and 91% of its volume between 1996 and 2016. One of the main factors that contributed to the drying up of Lake Urmia is the overexploitation of water resources due to the unsustainable development of agriculture, which the evidence is a 400% increase in the number of wells in the basin between 1984 and 2012 and a 60% increase in croplands development between 1987 and 2020^27^. The agricultural sector has played a vital role, accounting for almost 90% of the total water consumption in the basin^28,29^. Therefore, the indiscriminate expansion of agriculture can cause serious environmental impacts on the lake.

The paper formulates a framework based on probability and Artificial Intelligence (AI) modeling to delineate CSI for 12 medicinal plants within Lake Urmia. Most of the previous studies are based on expert judgments, and the present framework decreases the inherent subjectivities, as outlined as follows: (i) conducting frequency analysis to calculate the occurrence probability of desired precipitation and temperature, unlike the previous studies, which deterministically use subjective rate or expected values in the geospatial analysis; and (ii) overlaying the precipitation and temperature layers using the PSO algorithm as an unsupervised clustering technique to decrease the inherent subjectivity related to scoring systems. The results offer different cultivation options for managers and local farmers, giving them the opportunity to switch from their current cultivation practices. On the other hand, the unsustainable management of water resources in the agricultural sector has caused a crisis in the Lake Urmia basin, making it critical to apply the developed formulation in the area.

## Study area

**Fig. 1:**
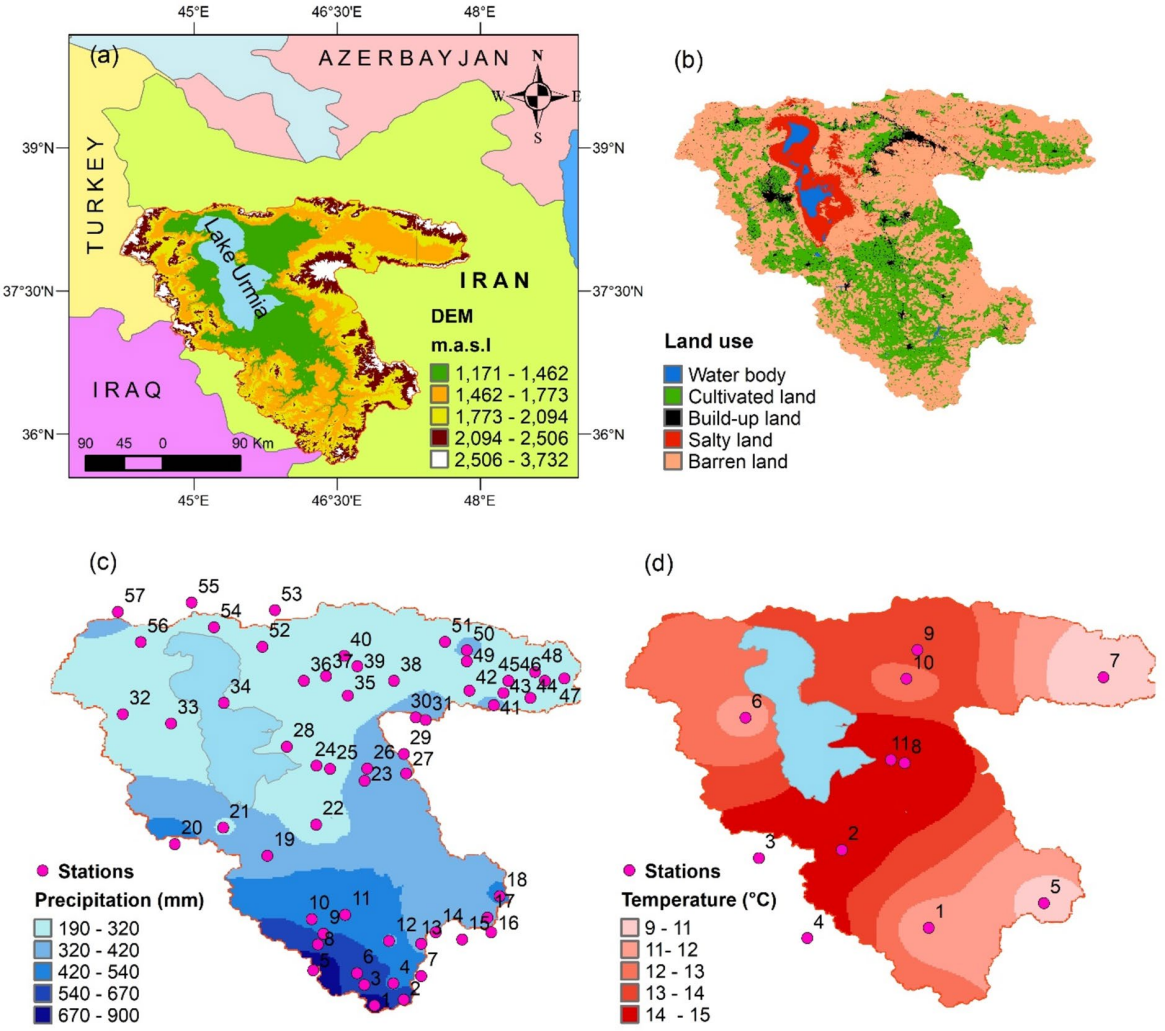
Study area: (**a**) geological location; (**b**) land use; (**c**) annual average precipitation in 2000–2020; (**d**) daily average temperature in 2000–2020. *Note* The figure is produced by the authors using QGIS 3.10.

The Lake Urmia basin, located in the northwest of Iran, has an approximate area of 51,876 km^2^, which occupies 3.2% of the total area of Iran. The basin is surrounded by mountains such as the southern slopes of Mount Sablan and the northern part of the Zagros Mountains, as well as the north, western, and southern slopes of Mount Sahand. Figure 1a shows the geographical location and altitude of the basin, varying from 1171 to 3700 m, considering the Digital Elevation Model (DEM) (SRTM-1 satellite in 2014). Figure 1b shows the land use map of the basin using Sentinel-2 satellite images from 2020 to 2022. The figure indicates that the study area was occupied by water bodies (1.8%), cultivated lands (28.7%), build-up lands (3.4%), salty lands (6.8%), and barren lands (59.3%).

Figure 1c,d show the spatial distributions for the annual precipitation in 57 stations and daily temperature in 11 stations in 2000–2020 obtained by the IDW interpolation technique. Notably, the data were provided by the Iran Meteorological Organization. The precipitation in Fig. 1c varies from 192 to 890 mm, with an average of 390 mm in 2000–2020. The figure indicates that precipitation decreases from the southwest parts to the north of the basin. The daily average temperature in Fig. 1d varies from 9 to 15 °C, with an average value of 12 °C. Based on average precipitation and temperature values, the climate of the basin is cold and semi-arid, as per Emberger (1930)^30^.

## Results

### Precipitation distributions

**Fig. 2:**
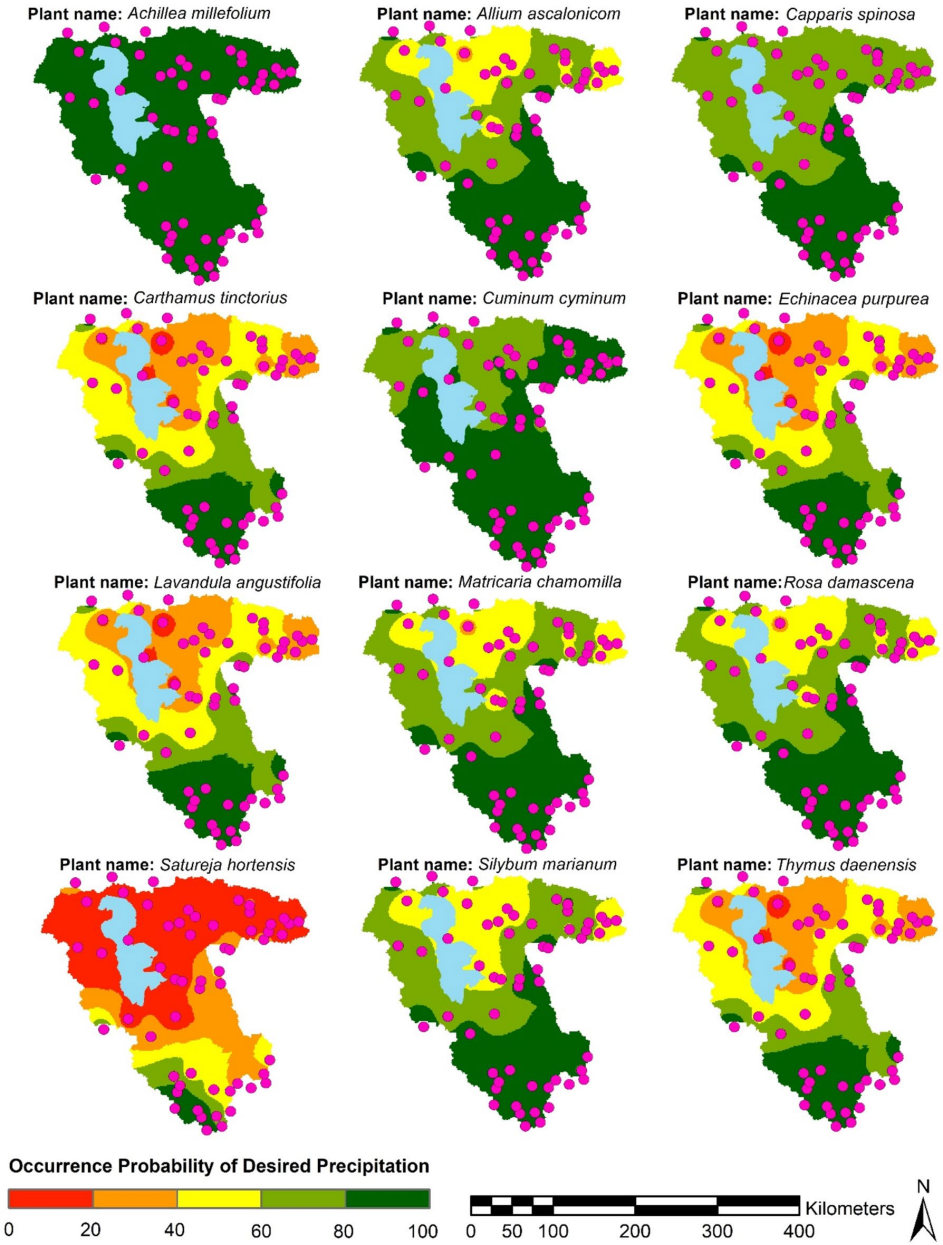
Probability occurrence values of desired precipitation. *Note* The figure is produced by the authors using QGIS 3.1.

Table S1 presents the goodness-of-fit results for the selected PDFs based on the Kolmogorov–Smirnov (K–S) test. Figure 2 depicts the spatial distribution of the probability of occurrence for desired annual precipitation, interpolated by IDW and classified into five bands. Notably, the occurrence probability values were calculated based on the frequency analyses by the fitted PDFs given in Table S1, considering the desired precipitation in Table 2. According to the figure, *Achillea millefmlant* can be cultivated throughout the basin. Also, the cultivation of *Allium ascalonicom*, *Matricaria chamomilla*, *Rosa damasena*, and *Silybum marianum* in the north and northeast of the basin has an average potential, but the potential increases towards the south. For *Capparis spinosa* and *Cuminum cyminum*, the south of the basin is located in areas with the highest potential, and the north is located in relatively high areas. *Echinacea purpurea*, *Carthamus tinctorius*, *Lavandula angustifolia*, and *Thymus daenensis* have the highest potential in the southern parts, but the potential decreases towards the north of the basin. For *Satureja hortensis*, the high potential part was only observed in the southwest parts of the basin.

**Table 2:** Investigated medicinal plants and desired precipitation.

Plant		Desired annual precipitation (mm)	Temperature	
			Desired value (°C)	Growth period
1	*Achillea millefolium*	120	18 to 26	Early April to late August
2	*Allium ascalonicom*	250	9 to 13	Late October to early July
3	*Capparis spinose*	200	− 8 to 50	Early March to early October
4	*Carthamus tinctorius*	300	15 to 20	Late March to late July
5	*Cuminum cyminum*	150	15 to 21	Late March to late July
6	*Echinacea purpurea*	300	18 to 26	Early March to early October
7	*Lavandula angustifolia*	300	18 to 25	Mid-April to late September
8	*Matericaria chamomilla*	140	15 to 19	Mid-April to late August
9	*Rosa damasena*	250	15 to 22	Early April to mid-July
10	*Satureja hortensis*	450	20 to 25	Mid-April to mid-September
11	*Silybum marianum*	240	18 to 20	Late March to mid-August
12	*Thymus daenensis*	300	18 to 21	Early May to late September

### Temperature distributions

**Fig. 3:**
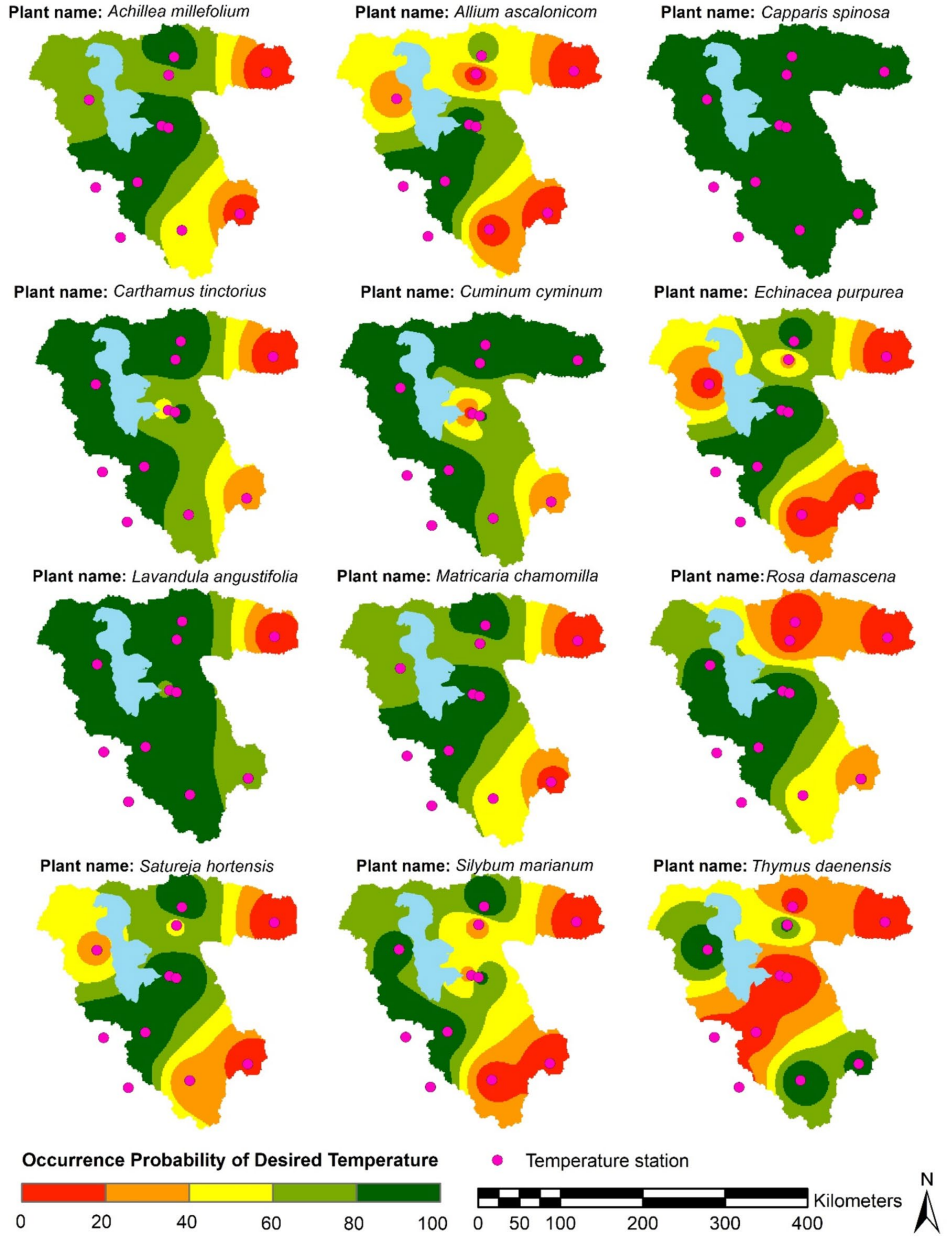
Probability occurrence values of desired temperature. *Note* The figure is produced by the authors using QGIS 3.1.

Figure 3 shows the spatial distribution of the occurrence probability of the desired temperature in the related growth period of each plant, which was interpolated by IDW and classified into five bands. Notably, a set of frequency analyses were conducted to calculate the occurrence probability using the fitted PDFs presented in Table S2, considering the desired temperature in Table 2. According to the figure, *Capparis spinosa* can be cultivated throughout the basin. Also, the cultivation of *Satureja hortensis*, *Matricaria chamomilla*, *Echinacea purpurea*, *Achillea millefmlant*, and *Silybum marianum* in the east of the basin has medium and weak potential. However, the potential increases towards the central and southwestern regions. *Carthamus tinctorius* and *Lavandula angustifolia* have the highest potential in the west and central regions, but the potential is low in the northeast. *Thymus daenensis* has the highest potential in the northeastern and southern areas, but the potential in the rest of the parts is medium and weak. For *Rosa damasena*, the central and western regions have the highest potential, and the potential decreases towards the northern and northeastern regions. The potential for *Cuminum cyminum* is quite high in the north and northwest and moderate from the central to the southeast. *Allium ascalonicom* has high potential in some parts of the west and central areas, but the potential is low in other areas.

### CSI distributions by PSO

**Fig. 4:**
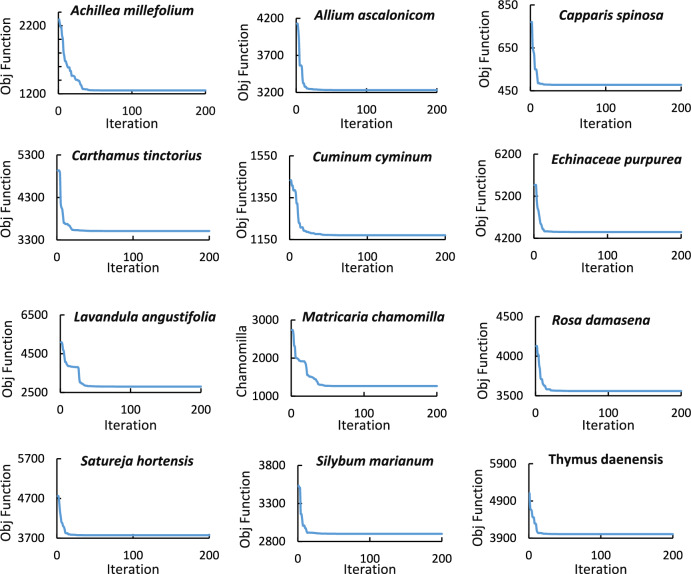
Objective function diagrams of PSO for clustering precipitation and temperature.

**Fig. 5:**
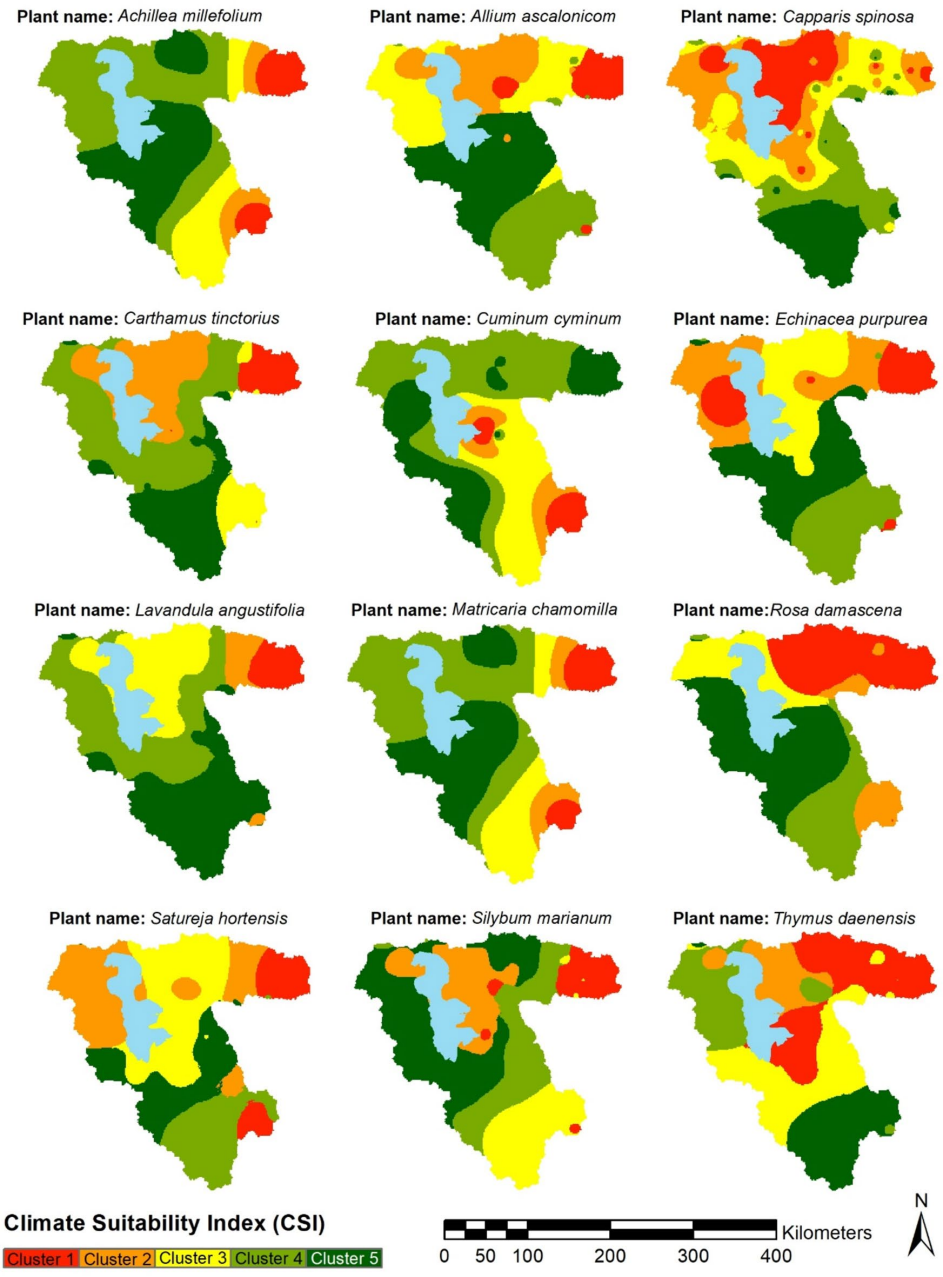
CSI for the investigated medicinal plants. *Note* The figure is produced by the authors using QGIS 3.1.

CSI values for the investigated medicinal plant were calculated using PSO to decrease the inherent subjectivity within expert judgment with the weights of precipitation and temperature layers. As an AI-based unsupervised clustering technique, PSO conducts cluster analysis to classify the study area into five bands by minimizing the objective function described in the method section. The PSO configuration includes a maximum iteration to complete the optimization process equal to 200, and the number of clusters is five. The constriction coefficients method was used to calculate the coefficients of inertia, in which two positive numbers were considered with a sum greater than four. Figure 4 depicts the objective function diagrams for each plant, which all converged, considering their tendency to the x-axis by increasing the iteration. After conducting cluster analysis, the precipitation and temperature layers were classified into five clusters using the PSO algorithm, and CSI clusters were distributed in Fig. 5 for each plant. Notably, CSI increases from Cluster 1 to 5, corresponding to the red-to-green color palette.

Figure 5 indicates that the CSI for *Achillea millefolium* and *Matricaria chamomilla* is low in the eastern regions and becomes high in the central and western regions. The CSI for *Allium ascalonicom* is low in the northern parts but increases from the south to the central parts. The CSI for *Capparis spinosa* is low in the north half of the basin, but it increases from the central parts to the south of the basin. *Carthamus tinctorius* has the lowest CSI in the areas around Lake Urmia and the eastern parts, but the higher CSI clusters exist in the south and southwest parts. The CSI for *Cuminum cyminum* is high in northern and western regions but gradually decreases towards the central and southeastern regions. *Echinacea purpurea* can be cultivated in the central and southern regions, but cultivation is limited to the northern parts of the basin. The CSI for *Lavandula angustifolia* is low in the northeastern regions but moderate in the northern regions and increases towards the southern parts. The CSI for *Rosa damasena* varies from the low clusters in the northeastern regions to the central and western regions. *Satureja hortensis* has lower CSI in the northeastern and southeastern regions, and the CSI in northern regions changes from low and moderate to high clusters towards the south. The CSI for *Silybum marianum* is unsuitable in the central and northeastern areas, but it gradually increases from the southeast to the northwest. *Thymus Daenensis* has a lower CSI cluster in the central and northeastern regions; it increases in the northwest and southern regions.

## Discussion

The potential of rainfed cultivation for 12 medicinal plants in the Lake Urmia basin can be one of the promising solutions for the ecological and economic future of the basin and the lake distressed by unsustainable use of water resources. Previous studies investigated the relationship between the decrease in the Lake Urmia level and the agriculture growth^27^. If policymakers and managers adopt the agricultural patterns based on the present and similar studies dealing with rainfed agriculture, the restoration of Lake Urmia is possible since the agriculture sector consumes almost 90% of water. Even with this, the developed framework is not confined to specific case studies and can be adjusted to various basins and regions. Its application in other case studies is imperative to address its vulnerabilities and enhance its efficacy.

The study employed precipitation and temperature historical records, but using projected climate variables under climate change scenarios can provide a deeper insight into the climate suitability index. Utilizing projected climate variables allows policymakers to explore adaptive strategies to mitigate the impacts of climate change. While previous studies^31^ have investigated these impacts on indices concerning cultivation potential, the innovation in the suggested formwork sets it apart from the goals of the current study.

The investigated medicinal plants are compatible with the climate condition of the basin and, additionally, can generate higher economic benefits for local farmers. However, climate suitability is just one aspect of achieving the goals of this study. It is important to address other perspectives, such as the social impacts of the local farmers’ change in cultivation type. The change in cultivation type is closely tied to the livelihoods of local farmers, so it is crucial to ensure more comprehensive knowledge. Certain plants, such as *Rosa damasena*, may pose challenges to the local farmers, and they are difficult to cultivate due to their blades. Moreover, changing the cultivation type practiced for many years may not bring enough economic benefits in the initial stages of cultivation. Therefore, governments need to provide financial support for such programs.

Pouladi et al.^32^ discussed some socio-hydrological barriers to the restoration of Lake Urmia. They declared that farmers experiencing financial problems were unsympathetic to the drying up or restoration of Lake Urmia. Changing the cultivation pattern in low economic conditions towards medicinal plants requires financial support for farmers because it will take time to benefit the farmer. The successful implementation of such decisions requires the participation of farmers, which can be achieved by understanding and reflecting their concerns in the decision-making process.

In addition to social aspects, economic and environmental aspects play significant roles in developing a comprehensive index for evaluating plant cultivation. When assessing plants for suitability, economic considerations such as market demand and value can be crucial in determining which species are prioritized for further investigation. By understanding the economic significance of these plants, policymakers can make informed decisions about resource allocation and conservation efforts. From an environmental standpoint, it is essential to recognize the potential impact of introduced plants on native species and ecosystems. Some plant species may exhibit invasive behavior, outcompeting native flora and disrupting the ecological balance. Therefore, careful evaluation of a plant's potential invasiveness is essential to safeguarding the biodiversity and stability of an area. Another environmental aspect to consider in plant evaluation is the quality and texture of the soil. Soil characteristics can significantly influence plants' growth and survival, affecting their overall suitability for a particular environment. By considering soil conditions, researchers can better understand the constraints and opportunities for plant species in a given habitat, leading to a more thorough and holistic assessment.

The cultivation of medicinal plants in the Lake Urmia basin is limited, and even sparse data is not available, making it difficult to verify the results directly. However, the goodness of fit of the fitted Probability Density Functions (PDFs) based on the K–S test confirms the accuracy of the results. It should be noted that errors due to interpolation may affect the results, but we have tried to manage this by making maximum use of the available precipitation stations. The number of precipitation stations is relatively adequate and covers the study area. Although the number of temperature stations is relatively low, gradual changes in temperature compared to precipitation make it acceptable.

The CSI is affected by climate variables obtained by data from point stations, and the IDW technique spatially distributes the point results. This procedure neglects microclimates between the stations, which arise from elevation, urban development, and proximity to bodies of water. This is regarded as the other limitation of present studies. The main sources of microclimate in the basin are Sahand Mountain in the western part of the basin and the water crisis in Lake Urmia. Previously, the large size of Lake Urmia played an influential role in regulating microclimates. However, as it continues to shrink, evidence shows that the daily temperature range has widened, humidity levels have decreased, and the annual precipitation pattern has shifted^33,34^. These variations provide room for future studies.

## Conclusion

The study developed a probabilistic framework for identifying the high-potential areas for rainfed cultivation of 12 medicinal plants based on precipitation and temperature. The study delineated the high-potential areas by calculating an index, referring to SCI. Two main components for SCI calculations include (i) climate variables, prepared by frequency analysis to calculate the occurrence probability of desired precipitation and temperature, and (ii) the combination of climate variables to calculate CSI, conducted by PSO as an unsupervised clustering technique. These components reduce the inherent subjectivity associated with expert judgment. We implemented this framework in the Lake Urmia basin, facing challenges from unsustainable water resources management. The study identified suitable areas for rainfed cultivation of each plant within the basin, which could help policymakers make informed decisions to enhance agriculture efficiency and preserve water resources. This breakthrough has the potential to transform the region's agriculture sector and set an example for other areas facing similar challenges. The limitations of the study provide fertile ground for future studies, including microclimate changes due to Lake Urmia dryness, the interpolation error due to microclimate changes between stations, the impact of climate change on CSI, and the incorporation of social, economic, and environmental aspects of the investigated plants within the framework.

## Method

**Fig. 6:**
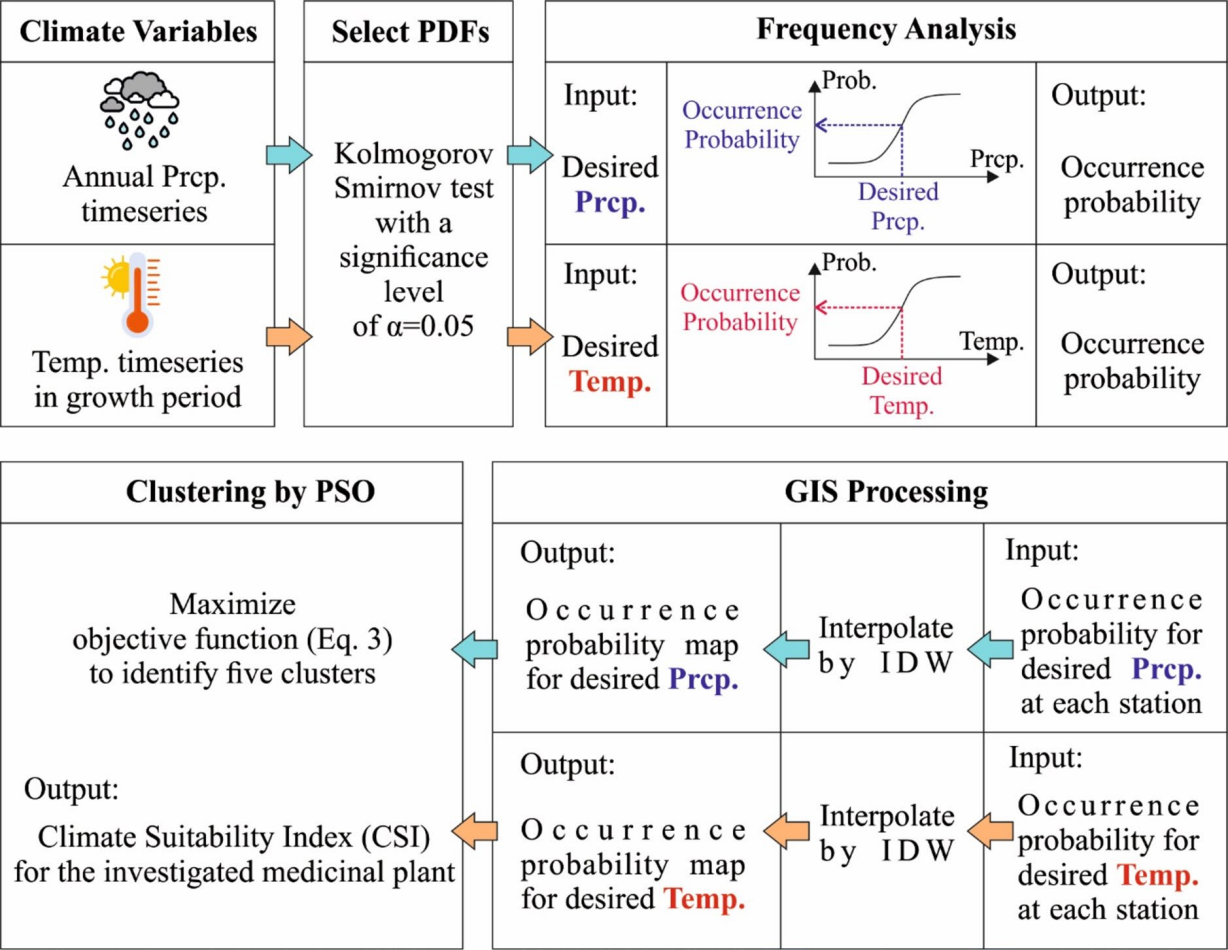
Methodological flowchart of the probabilistic framework for mapping CSI.

This study formulates a probabilistic framework for mapping CSI of 12 medicinal plants using climate variables of precipitation and temperature. Figure 6 shows the methodological flowchart of the framework, and the essential components outlined as follows: (i) data pre-processing; (ii) fitting appropriate Probability Distribution Functions (PDFs) to precipitation and temperature data at each station considering the desired precipitation and temperature for each plant (see Table 2); (iii) and conducting the frequency analysis for calculating the occurrence probability for the desired precipitation and temperature; (iv) spatially distributing the calculated occurrence probabilities of precipitation and temperature for each plant; and (iv) clustering precipitation and temperature layers for each plant by the PSO algorithm.

### Pre-processing

The outlier test and the gap data estimation were conducted in the pre-processing stage. Outlier data were detected using the interquartile range method^35^. Also, gap data were estimated by fitting the regression equations between stations with gap data and the station having the highest correlation coefficient.

### Precipitation layers

Precipitation in rainfed and irrigated cultivations plays a vital role in increasing the efficiency of agriculture. The medicinal plants were selected based on their adaptability in the cold and semi-dry climate of the Lake Urmia basin, as well as their optimal water requirement during the growth period. Table 2 presents the investigated plants and corresponding desired annual precipitation values and desired temperature corresponding to the growth periods, which are collected referring to propagation books published by the Ministry of Agriculture Jihad.

### Temperature layers

Temperature is crucial for calculating the CSI of medicinal plants, and desired values are required during the growth period. Table 2 presents the growth period and the corresponding desired temperature refereeing propagation books published by the Ministry of Agriculture Jihad. Notably, the Capparis Spinosa has deep and extensive roots and can tolerate severe summers in areas with low precipitation and temperatures of 50 °C. This plant not only shows considerable resistance to water shortage and high temperatures, but it is also resistant to cold and can survive up to − 8 °C^36^.

### Goodness-of-fit for selecting PDFs

The precipitation and temperature data were extracted at each station, and a broad set of PDFs were fitted to the extracted data. The goodness-of-fit test for the fitted PDFs was conducted in the study by the K–S test^37^. Then, the appropriate PDFs were accepted in this test with a significance level of α = 0.05.

### Spatial interpolation

The study employed the Inverse Distance Weighted (IDW) technique to spatially interpolate the probability occurrence of desired precipitation and temperature at different stations. IDW uses the neighboring points by averaging the sample points located in the neighborhood of each unknown point. Also, IDW assumes that the effect of each point is proportional to the power of the inverse of its distance, so the effect of the known point decreases with increasing distance^38^.

### Artificial intelligence model

As a AI-based algorithm, PSO was first introduced by Kennedy et al.^39^. PSO has the capability to optimize non-linear problems with immediate convergence rate and a relatively low calculation burden. The design of this algorithm is inspired by the movement of flocks and fish to optimize the path to food^40^. In PSO, each community member is called a particle, and the position of each particle is a potential solution to the optimization problem. The position of the particles changes according to their experience that of their neighbors during the execution of the algorithm, and thus, the particles float and move in the search space. As the particles move, each stores a memory of its best value and corresponding position along its path. The value of the particles is measured according to the criterion function related to the optimization problem. For the *i*^th^ particle, the best value and its corresponding position are stored in the variables ${pbest}_{i}$ and ${p}_{i}=({p}_{i1}.{p}_{i2}. \dots .{p}_{iD})$, respectively. Notably, *D* shows the dimension of the search space. Also, the best particle position in all iterations and its corresponding position are stored in the same variable. The collection of *n* particles in a community is called population and is expressed as $pop=({p}_{1}.{p}_{2}. \dots .{p}_{n})$^37^. The basis of the PSO method is based on the principles accelerating towards their ${pbest}_{i}$ and ${gbest}_{i}$ in each iteration, with the addition of the displacement vector in each particle, a new position in the search space for each particle is found. At the end of the algorithm, the position corresponding to ${gbest}_{i}$ is considered as the optimal solution of the problem. In each iteration, the position and velocity of the i^th^ particle are updated according to Eqs. (1) and (2):

1 $$v_{i}^{iter + 1} = w v_{id}^{iter} + c_{1} rand{\text{\rm O}}\left( {p_{id} - x_{id}^{iter} } \right) + c_{2} rand{\text{\rm O}}\left( {p_{gd} - x_{id}^{iter} } \right)$$

2 $$x_{id}^{iter + 1} = x_{id}^{iter} + V_{id}^{iter + 1}$$

where ${V}_{i}=({v}_{i1}.{v}_{i2}.\dots .{v}_{id})$ is the velocity vector of the *i*th particle; and ${X}_{i}=({x}_{i1}.{x}_{i2}. \dots .{x}_{iD})$ is its position vector. In this regard, *c*_*1*_ and *c*_*2*_ are positive numbers called personal and global learning coefficient, respectively. The *randΟ* is a function that generates random numbers between zero and one. *w* is the inertia factor that controls the effect of the current speed on the current speed, and its allowed value in the standard PSO algorithm changes in the range of 0.4 to 0.9.

The study transfers the clustering problem into the optimization problem by defining the objective function and connecting it to the PSO algorithm. The objective function is defined by minimizing the distances of each data from its cluster center, i.e., within-cluster distance by Eq. (3):

3 $$Obj \, Function = min\mathop \sum \limits_{j = 1}^{k} \mathop \sum \limits_{i = 1}^{n} d\left( {x_{i} .m_{j} } \right)$$

where *x*_*i*_ is a member of the total data of $X=\left\{{x}_{1}.{x}_{2}.\dots .{x}_{n}\right\}$; *m*_*j*_ is a member of the cluster centers as $X=\left\{{x}_{1}.{x}_{2}.\dots .{x}_{n}\right\}$; and the *d* represents the Euclidian distance.

### Supplementary Information

Supplementary Tables.

## Data Availability

Data are available from the corresponding author upon reasonable request.
