# Supplementary material for: Mapping climate suitability index for rainfed cultivation of medicinal plants by developing an AI-based probabilistic framework
Source: Sci Rep. 2024 Sep 2;14:20413. doi: 10.1038/s41598-024-71208-6 (PMC11369235; doi:10.1038/s41598-024-71208-6)
Supplement: Supplementary file 1 — Supplementary Tables. [file 41598_2024_71208_MOESM1_ESM.docx]

**Supplementary Material**

**Table S1**. Statistical characteristics of precipitation data and the result of goodness-of-fit with significance level of α=0.05 for selected PDFs

| **Station** | **Annual Precipitation** | | | | | **Station** | **Annual Precipitation** | | | | |
| --- | --- | --- | --- | --- | --- | --- | --- | --- | --- | --- | --- |
|  | **Mean**  **(mm)** | **Std**  **dev** | **PDF** | **For α=0.05** | |  | **Mean**  **(mm)** | **Std**  **dev** | **PDF** | **For α=0.05** | |
|  |  |  |  | **Statistics** | **Critical** |  |  |  |  | **Statistics** | **Critical** |
| 1 | 787.8 | 171.9 | Wakeby | 0.09 | 0.28 | 30 | 341.6 | 82.7 | Burr | 0.1 | 0.29 |
| 2 | 637.3 | 345.9 | Gen.logistic | 0.1 | 0.28 | 31 | 422.1 | 96.1 | Gen.Extreme value | 0.1 | 0.29 |
| 3 | 665.5 | 136.1 | Gen.Extreme | 0.11 | 0.28 | 32 | 308.5 | 105 | Weibull | 0.09 | 0.28 |
| 4 | 500 | 99.9 | beta | 0.09 | 0.28 | 33 | 302.9 | 79.2 | Nakagami | 0.09 | 0.28 |
| 5 | 960.5 | 201.3 | Frechet(3p) | 0.1 | 0.28 | 34 | 242.6 | 59 | Log-normal | 0.13 | 0.3 |
| 6 | 622.7 | 144.1 | Gen.Pareto | 0.09 | 0.28 | 35 | 292.7 | 54.5 | Triangular | 0.11 | 0.29 |
| 7 | 429.7 | 84.1 | Log-Pearson3 | 0.08 | 0.28 | 36 | 244.6 | 67.3 | Pearson6(4p) | 0.08 | 0.29 |
| 8 | 661.3 | 157.4 | LogGamma | 0.12 | 0.28 | 37 | 248.7 | 58.1 | JohnsonSB | 0.09 | 0.29 |
| 9 | 510.7 | 108.4 | Error | 0.09 | 0.28 | 38 | 289.4 | 70.3 | Frechet | 0.15 | 0.29 |
| 10 | 512.2 | 138.4 | Wakeby | 0.09 | 0.28 | 39 | 192.6 | 89.3 | JohnsonSB | 0.11 | 0.28 |
| 11 | 434 | 98.7 | Pearson6(4p) | 0.07 | 0.28 | 40 | 259.1 | 54.3 | Nakagami | 0.1 | 0.28 |
| 12 | 411.3 | 93.4 | Beta | 0.07 | 0.28 | 41 | 401.5 | 172.2 | Beta | 0.09 | 0.29 |
| 13 | 408.2 | 96.3 | Nakagami | 0.07 | 0.28 | 42 | 258 | 66.9 | Frechet(3p) | 0.15 | 0.29 |
| 14 | 398.2 | 93.5 | Log-Pearson3 | 0.06 | 0.28 | 43 | 269.1 | 66.1 | Nakagami | 0.08 | 0.29 |
| 15 | 289.5 | 71.7 | Wakeby | 0.07 | 0.28 | 44 | 241.5 | 45.1 | Wakeby | 0.11 | 0.29 |
| 16 | 372.9 | 95.7 | Wakeby | 0.1 | 0.29 | 45 | 293.6 | 56.59 | Dagum | 0.07 | 0.29 |
| 17 | 317.4 | 103.8 | Nakagami | 0.08 | 0.29 | 46 | 264.1 | 64.5 | Wakeby | 0.12 | 0.29 |
| 18 | 447 | 104.3 | Gen.Extreme value | 0.09 | 0.28 | 47 | 256.6 | 67.7 | JohnsonSB | 0.07 | 0.29 |
| 19 | 372.1 | 91.4 | Log-Logistic | 0.12 | 0.28 | 48 | 242 | 56.3 | JohnsonSB | 0.11 | 0.29 |
| 20 | 522.7 | 141.5 | Gen.Logistic | 0.09 | 0.28 | 49 | 264.3 | 78.9 | Wakeby | 0.12 | 0.29 |
| 21 | 314.7 | 91 | Wakeby | 0.09 | 0.28 | 50 | 358.4 | 86.9 | Gamma | 0.08 | 0.3 |
| 22 | 272.6 | 75.6 | Beta | 0.12 | 0.28 | 51 | 294.5 | 98.1 | Pearson5 | 0.1 | 0.3 |
| 23 | 399.1 | 113.2 | Error | 0.11 | 0.29 | 52 | 225 | 55.3 | Hypersecant | 0.11 | 0.28 |
| 24 | 262.8 | 75.5 | Wakeby | 0.08 | 0.28 | 53 | 270 | 66.7 | Gen.Logistic | 0.1 | 0.3 |
| 25 | 254.8 | 64.4 | Gen.Extreme value | 0.07 | 0.28 | 54 | 257.3 | 74.6 | JohnsonSB | 0.1 | 0.29 |
| 26 | 330.2 | 100.1 | Pearson5 | 0.09 | 0.29 | 55 | 327.9 | 82 | Burr | 0.11 | 0.29 |
| 27 | 373.6 | 107.3 | Cauchy | 0.1 | 0.29 | 56 | 234.9 | 67.5 | Wakeby | 0.08 | 0.28 |
| 28 | 235 | 65.2 | Gen.Logistic | 0.07 | 0.28 | 57 | 409.7 | 129.1 | Wakeby | 0.1 | 0.28 |
| 29 | 37.45 | 46.65 | Gen.Extreme | 0.1 | 0.29 |  |  |  |  |  |  |

**Table S2**. Statistical characteristics of temperature data and the result of goodness-of-fit with significance level of α=0.05 for selected PDFs

| St. | Mean  (mm) | Std.  Dev. | PDF | For α=0.05 | | Mean  (mm) | Std.  Dev. | PDF | For α=0.05 | |
| --- | --- | --- | --- | --- | --- | --- | --- | --- | --- | --- |
|  |  |  |  | Statistics | Critical |  |  |  | Statistics | Critical |
| ***Achillea millefolium*** | | | | | | ***Allium ascalonicom*** | | | | |
| 1 | 18.1 | 0.78 | Wakeby | 0.07 | 0.28 | 7.7 | 0.91 | Weibull | 0.10 | 0.28 |
| 2 | 20.2 | 0.90 | Uniform | 0.10 | 0.28 | 9.7 | 0.88 | Gen.Extreme value | 0.07 | 0.28 |
| 3 | 20.4 | 0.72 | Hypersecant | 0.12 | 0.28 | 9.7 | 0.92 | Weibull(3p) | 0.10 | 0.28 |
| 4 | 20.4 | 0.81 | Wakeby | 0.09 | 0.28 | 9.8 | 0.80 | Log-Logistic(3p) | 0.08 | 0.28 |
| 5 | 17 | 0.81 | LogGamma | 0.10 | 0.28 | 6.2 | 0.82 | Inv.Gaussian | 0.07 | 0.28 |
| 6 | 18.5 | 0.77 | JohnsonSB | 0.09 | 0.28 | 8.1 | 0.86 | Inv.Gaussian | 0.09 | 0.28 |
| 7 | 15.7 | 1.08 | Burr(4p) | 0.08 | 0.28 | 5.3 | 1.00 | Cauchy | 0.11 | 0.28 |
| 8 | 20.5 | 0.78 | Cauchy | 0.12 | 0.28 | 9 | 0.96 | Gen.Extreme value | 0.11 | 0.28 |
| 9 | 20.3 | 0.75 | Wakeby | 0.14 | 0.28 | 9.1 | 0.92 | JohnsonSB | 0.07 | 0.28 |
| 10 | 18.7 | 0.86 | JohnsonSB | 0.11 | 0.28 | 8.1 | 0.92 | Burr | 0.08 | 0.28 |
| 11 | 22.4 | 1.22 | Kumaraswamy | 0.09 | 0.28 | 11 | 0.90 | Cauchy | 0.12 | 0.28 |
| ***Capparis spinosa*** | | | | | | ***Carthamus tinctorius*** | | | | |
| 1 | 16.7 | 10 | Wakeby | 0.08 | 0.28 | 15.5 | 0.75 | Gen.Extreme value | 0.08 | 0.28 |
| 2 | 18.8 | 11 | Wakeby | 0.08 | 0.28 | 17.8 | 0.86 | JohnsonSB | 0.07 | 0.28 |
| 3 | 19 | 0.69 | Wakeby | 0.11 | 0.28 | 17.8 | 0.70 | JohnsonSB | 0.10 | 0.28 |
| 4 | 19 | 0.72 | Wakeby | 0.09 | 0.28 | 17.6 | 0.79 | JohnsonSB | 0.09 | 0.28 |
| 5 | 15.5 | 0.70 | Wakeby | 0.11 | 0.28 | 14.3 | 0.79 | Pearson6(4p) | 0.11 | 0.28 |
| 6 | 17 | 0.71 | Wakeby | 0.09 | 0.28 | 16.2 | 0.76 | JohnsonSB | 0.07 | 0.28 |
| 7 | 14.1 | 0.89 | Wakeby | 0.09 | 0.28 | 13.2 | 0.78 | Gen.Logistic | 0.09 | 0.28 |
| 8 | 19 | 0.72 | Wakeby | 0.07 | 0.28 | 17.8 | 0.78 | Gen.Logistic | 0.09 | 0.28 |
| 9 | 18.8 | 0.66 | Wakeby | 0.08 | 0.28 | 17.7 | 0.76 | Gen.Logistic | 0.12 | 0.28 |
| 10 | 17.3 | 0.77 | Wakeby | 0.08 | 0.28 | 16.2 | 0.83 | JohnsonSB | 0.09 | 0.28 |
| 11 | 20.9 | 1.16 | Wakeby | 0.07 | 0.28 | 19.8 | 0.99 | Gen.Logistic | 0.08 | 0.28 |
| ***Cuminum cyminum*** | | | | | | ***Echinacea purpurea*** | | | | |
| 1 | 15.5 | 0.75 | Gen.Extreme value | 0.08 | 0.28 | 16.7 | 0.66 | Error | 0.07 | 0.28 |
| 2 | 17.8 | 0.86 | JohnsonSB | 0.07 | 0.28 | 18.8 | 0.74 | Wakeby | 0.08 | 0.28 |
| 3 | 17.8 | 0.70 | JohnsonSB | 0.10 | 0.28 | 19 | 0.69 | Gen.Logistic | 0.07 | 0.28 |
| 4 | 17.6 | 0.79 | JohnsonSB | 0.09 | 0.28 | 19 | 0.73 | JohnsonSB | 0.10 | 0.28 |
| 5 | 14.4 | 0.82 | Error | 0.11 | 0.28 | 15.5 | 0.70 | PowerFunc | 0.11 | 0.28 |
| 6 | 16.2 | 0.76 | JohnsonSB | 0.07 | 0.28 | 17 | 0.71 | JohnsonSB | 0.07 | 0.28 |
| 7 | 13.2 | 0.78 | Gen.Logistic | 0.09 | 0.28 | 14.1 | 0.90 | Burr | 0.09 | 0.28 |
| 8 | 17.4 | 1.46 | Wakeby | 0.08 | 0.28 | 19 | 0.72 | GenExtreme | 0.09 | 0.28 |
| 9 | 17.7 | 0.76 | JohnsonSB | 0.09 | 0.28 | 18.8 | 0.66 | JohnsonSB | 0.11 | 0.28 |
| 10 | 16.1 | 0.83 | GenPareto | 0.09 | 0.28 | 17.3 | 0.78 | Error | 0.11 | 0.28 |
| 11 | 19.8 | 0.99 | Gen.Logistic | 0.08 | 0.28 | 20.9 | 1.17 | Wakeby | 0.09 | 0.28 |
| ***Lavandula angustifolia*** | | | | | | ***Matricaria chamomilla*** | | | | |
| 1 | 19.6 | 0.73 | Gen.Extreme value | 0.07 | 0.28 | 19.2 | 0.78 | Dagum | 0.08 | 0.28 |
| 2 | 21.7 | 0.79 | Burr | 0.09 | 0.28 | 21.3 | 0.85 | Burr | 0.11 | 0.28 |
| 3 | 21.7 | 0.85 | Gen.Logistic | 0.08 | 0.28 | 21.5 | 0.65 | Burr | 0.09 | 0.28 |
| 4 | 22.1 | 0.83 | Wakeby | 0.09 | 0.28 | 21.6 | 0.81 | Wakeby | 0.06 | 0.28 |
| 5 | 18.5 | 0.76 | Gamma (3p) | 0.08 | 0.28 | 18.2 | 0.82 | Erlang(3p) | 0.10 | 0.28 |
| 6 | 19.8 | 0.74 | Gen.Extreme value | 0.08 | 0.28 | 19.6 | 0.77 | JohnsonSB | 0.07 | 0.28 |
| 7 | 17 | 1.20 | Dagum | 0.10 | 0.28 | 14.5 | 0.88 | Wakeby | 0.11 | 0.28 |
| 8 | 22 | 0.78 | Wakeby | 0.08 | 0.28 | 21.6 | 0.80 | Wakeby | 0.08 | 0.28 |
| 9 | 21.9 | 0.71 | Pert | 0.09 | 0.28 | 21.5 | 0.73 | Wakeby | 0.09 | 0.28 |
| 10 | 20.3 | 0.83 | Wakeby | 0.06 | 0.28 | 19.8 | 0.86 | Wakeby | 0.12 | 0.28 |
| 11 | 24 | 1.37 | Wakeby | 0.10 | 0.28 | 23.6 | 1.28 | JohnsonSB | 0.12 | 0.28 |

**Table S2 (Continued)**. Statistical characteristics of temperature data and the result of goodness-of-fit with significance level of α=0.05 for selected PDFs

| St. | Mean  (mm) | Std.  Dev. | PDF | For α=0.05 | | Mean  (mm) | Std.  Dev. | PDF | For α=0.05 | |
| --- | --- | --- | --- | --- | --- | --- | --- | --- | --- | --- |
|  |  |  |  | Statistics | Critical |  |  |  | Statistics | Critical |
| ***Rosa damasena*** | | | | | | ***Satureja hortensis*** | | | | |
| 1 | 15 | 0.77 | Pearson5(3p) | 0.07 | 0.28 | 19.6 | 0.76 | Log-Pearson3 | 0.08 | 0.28 |
| 2 | 17.4 | 0.90 | Wakeby | 0.06 | 0.28 | 21.7 | 0.82 | Gen.Extreme value | 0.10 | 0.28 |
| 3 | 17.4 | 0.73 | Wakeby | 0.12 | 0.28 | 21.9 | 0.70 | Gamma (3p) | 0.09 | 0.28 |
| 4 | 17.2 | 0.82 | Wakeby | 0.08 | 0.27 | 22 | 0.79 | Wakeby | 0.07 | 0.28 |
| 5 | 14 | 0.87 | Wakeby | 0.08 | 0.28 | 18.5 | 0.79 | Wakeby | 0.07 | 0.28 |
| 6 | 15.9 | 0.77 | Gen.Pareto | 0.10 | 0.28 | 19.7 | 0.79 | Gen.Extreme value | 0.08 | 0.28 |
| 7 | 13 | 0.77 | LogPearson3 | 0.10 | 0.28 | 18.6 | 0.90 | Dagum | 0.12 | 0.28 |
| 8 | 17.4 | 0.80 | LogPearson3 | 0.08 | 0.28 | 22 | 0.78 | Hypersecant | 0.12 | 0.28 |
| 9 | 17.3 | 0.80 | Gen.Extreme value | 0.07 | 0.28 | 21.8 | 0.72 | Gen.Extreme value | 0.09 | 0.28 |
| 10 | 15.8 | 0.84 | JohnsonSB | 0.08 | 0.28 | 20.2 | 0.84 | JohnsonSB | 0.10 | 0.28 |
| 11 | 19.5 | 0.94 | Wakeby | 0.08 | 0.28 | 24 | 1.36 | Dagum | 0.10 | 0.28 |
| ***Silybum marianum*** | | | | | | ***Thymus daenensis*** | | | | |
| 1 | 16.5 | 0.78 | Beta | 0.07 | 0.28 | 18.9 | 0.61 | Gen.Extreme value | 0.08 | 0.28 |
| 2 | 18.6 | 0.94 | Gen.Extreme value | 0.09 | 0.29 | 20.8 | 0.74 | Burr | 0.09 | 0.28 |
| 3 | 18.8 | 0.73 | Wakeby | 0.08 | 0.28 | 21 | 0.89 | Gen.Logistic | 0.09 | 0.28 |
| 4 | 18.7 | 0.79 | Gen.Pareto | 0.10 | 0.28 | 21.9 | 0.91 | Wakeby | 0.09 | 0.28 |
| 5 | 15.4 | 0.82 | Erorr | 0.07 | 0.28 | 18.1 | 0.62 | Gamma (3p) | 0.06 | 0.28 |
| 6 | 17.1 | 0.79 | Gen.Extreme value | 0.10 | 0.28 | 19.3 | 0.59 | Gen.Extreme value | 0.10 | 0.28 |
| 7 | 14 | 0.85 | Dagum | 0.11 | 0.28 | 16.4 | 0.97 | Dagum | 0.10 | 0.28 |
| 8 | 18.8 | 0.81 | Gen.Logistic | 0.12 | 0.28 | 21.6 | 0.87 | Wakeby | 0.07 | 0.28 |
| 9 | 18.7 | 0.79 | Wakeby | 0.15 | 0.28 | 21.3 | 0.66 | Wakeby | 0.06 | 0.28 |
| 10 | 17.1 | 0.88 | Triangular | 0.08 | 0.28 | 19.5 | 0.91 | Pert | 0.09 | 0.28 |
| 11 | 20.8 | 1.11 | Gen.Extreme value | 0.13 | 0.28 | 23.4 | 1.26 | Wakeby | 0.09 | 0.28 |
